# Salidroside orchestrates metabolic reprogramming by regulating the Hif-1α signalling pathway in acute mountain sickness

**DOI:** 10.1080/13880209.2021.1992449

**Published:** 2021-11-05

**Authors:** Xiaoning Yan, Jie Liu, Meixia Zhu, Lirong Liu, Yijun Chen, Yinhuan Zhang, Menghan Feng, Zhixin Jia, Hongbin Xiao

**Affiliations:** a School of Chinese Materia Medica, Beijing University of Chinese Medicine, Beijing, China; b Research Center of Chinese Medicine Analysis and Transformation, Beijing University of Chinese Medicine, Beijing, China

**Keywords:** *Rhodiola crenulata*, lactate, glycolysis, oxidative phosphorylation

## Abstract

**Context:**

*Rhodiola crenulata* (Hook. f. et Thoms.) H. Ohba (Crassulaceae) is used to prevent and treat acute mountain sickness. However, the mechanisms underlying its effects on the central nervous system remain unclear.

**Objective:**

To investigate the effect of *Rhodiola crenulata* on cellular metabolism in the central nervous system.

**Materials and methods:**

The viability and Hif-1α levels of microglia and neurons at 5% O_2_ for 1, 3, 5 and 24 h were examined. We performed the binding of salidroside (Sal), rhodiosin, tyrosol and *p*-hydroxybenzyl alcohol to Hif-1α, Hif-1α, lactate, oxidative phosphorylation and glycolysis assays. Forty male C57BL/6J mice were divided into control and Sal (25, 50 and 100 mg/kg) groups to measure the levels of Hif-1α and lactate.

**Results:**

Microglia sensed low oxygen levels earlier than neurons, accompanied by elevated expression of Hif-1α protein. Salidroside, rhodiosin, tyrosol, and *p*-hydroxybenzyl alcohol decreased BV-2 (IC_50_=1.93 ± 0.34 mM, 959.74 ± 10.24 μM, 7.47 ± 1.03 and 8.42 ± 1.63 mM) and PC-12 (IC_50_=6.89 ± 0.57 mM, 159.28 ± 8.89 μM, 8.65 ± 1.20 and 8.64 ± 1.42 mM) viability. They (10 μM) reduced Hif-1α degradation in BV-2 (3.7-, 2.5-, 2.9- and 2.5-fold) and PC-12 cells (2.8-, 2.8-, 2.3- and 2.0-fold) under normoxia. Salidroside increased glycolytic capacity but attenuated oxidative phosphorylation. Salidroside (50 and 100 mg/kg) treatment increased the protein expression of Hif-1α and the release of lactate in the brain tissue of mice.

**Conclusions:**

These results suggest that Sal induces metabolic reprogramming by regulating the Hif-1α signalling pathway to activate compensatory responses, which may be the core mechanism underlying the effect of *Rhodiola crenulata* on the central nervous system.

## Introduction

*Rhodiola crenulata* (Hook. f. et Thoms.) H. Ohba (Crassulaceae) has a long history of widespread use as a botanical medicine in Europe, Asia and the United States to prevent and treat various common conditions and complex diseases, including acute mountain sickness (AMS) (Yi et al. 2015), fatigue (Shevtsov et al. 2003) and Alzheimer’s disease (Wang et al. 2017). Several studies have indicated that *Rhodiola crenulata* prevents and treats AMS by regulating the hypoxia-inducible factor-1 (Hif-1) signalling pathway (Wang et al. 2019). Salidroside (Sal), rhodiosin, tyrosol and *p*-hydroxybenzyl alcohol, the major functional ingredients of *Rhodiola crenulata*, may form a material basis for its anti-hypoxic effects (Chang et al. 2007, 2018; Liang et al. 2018; Yan et al. 2018). However, the active compounds and the exact role of *Rhodiola crenulata* in AMS remain unclear.

Salidroside possesses neuroprotective and cardioprotective effects with prominent antioxidative stress injury, anti-apoptosis and promotion of neurogenesis (Zhang et al. 2007, 2012; Liu et al. 2020). In addition, Sal stimulates hypoxia-inducible factor-1 alpha (Hif-1α) and erythropoietin (EPO) production to enhance neuronal survival under hypoxia and anti-inflammatory activities after cerebral ischaemia (Villa et al. 2003; Wei et al. 2017). Studies with cell experiments have shown that Sal stimulates the accumulation of Hif-1α protein via the reduction of Hif-1α degradation to provide an anti-hypoxic effect in kidney and liver cells (Zheng et al. 2012). However, the effect of Sal on microglia and neurons for its adaptogenic function remains unknown.

If the central nervous system is damaged, the brain is sensitive to hypoxia, including brain cell injury and brain oedema (Erecińska and Silver 2001). The adaptogenic activity of *Rhodiola crenulata* appears to be associated with a shift in metabolism during cold-hypoxia-restraint exposure and post-stress recovery (Gupta et al. 2008). Salidroside protects against myocardial injury from inflammation by regulating energy metabolism (Chang et al. 2016). Studies have shown that activating compensatory responses of astrocytes decreases aerobic oxidation and oxygen consumption but increases glycolysis (Marrif and Juurlink 1999; Vangeison et al. 2008). Hif-1α, the main transcriptional regulator of glycolysis, has been reported to reduce oxygen consumption, diminish the efficiency of oxidative phosphorylation (OXPHOS) and increase the dependency on glycolysis as an energy source (Iyer 1998; Gupta et al. 2009). Few studies have focussed on metabolic changes after treatment with active compounds of *Rhodiola crenulata* in the central nervous system.

Our study aimed to gain new insights into the active ingredients of *Rhodiola crenulata* against AMS. We hypothesized that active compounds of *Rhodiola crenulata* activate Hif-1 signalling and alter cellular metabolism to activate compensatory responses in the central nervous system. The present study used normoxia/hypoxia cellular models with 5% O_2_ or 21% O_2_ *in vitro*. We employed mouse microglia (BV-2), pheochromocytoma (PC-12) and C57BL/6J mice to clarify the mechanism of this compensatory function stimulated by *Rhodiola crenulata* in advance under normoxia or mild hypoxia.

## Materials and methods

### Chemicals and reagents

Dried *Rhodiola crenulata* was purchased from Tongrentang Store (Beijing, China) and identified by Professor Xueyong Wang (School of Chinese Materia Medica, Beijing University of Chinese Medicine, Beijing, China). Salidroside (H-040-171219), rhodiosin (H-070-171211), tyrosol (L-042-170426) and *p*-hydroxybenzyl alcohol (D-059-171216) were purchased from Chengdu Herbpurify Co., Ltd. (purity >98%, Chengdu, China). HPLC-grade acetonitrile and formic acid were purchased from Fisher Scientific (Fair Lawn, NJ). Ultra-pure water was freshly prepared using a Milli-Q system (Millipore, Milford, MA). A cell counting kit-8 (CCK-8) was purchased from Dojindo (Kumamoto, Japan). Dimethyl sulphoxide (DMSO), glucose (G7528), L-glutamine (25030-081) and sodium pyruvate (S8636) were purchased from Sigma-Aldrich (St. Louis, MO). The cell lysis buffer and prestained dual colour protein molecular weight markers for western blotting were obtained from Beyotime (Shanghai, China). Enhanced chemiluminescence (ECL) reagent was purchased from Merck Millipore (Darmstadt, Germany). The anti-Hif-1α (ab179483, 1:2000 dissolution) and anti-β-actin (ab179467, 1:5000 dissolution) were purchased from Abcam Technology (London, UK). Anti-rabbit IgG and horseradish peroxidase (HRP)-linked antibodies (#7074, 1:3000 dissolution) were purchased from Cell Signaling Technology (Boston, MA). The XF Cell Mito Stress Test Kit (103015-100), XFe24 FluxPak (102340-100), XF24 V7 PS cell culture microplates (100777-004), XF Calibrant (100840-000) and XF Base Medium (102353-100) were obtained from Agilent Seahorse Bioscience (Boston, MA).

### Preparations of reference substances and samples

Reference substances of *p*-hydroxybenzyl alcohol, Sal, tyrosol and rhodiosin were mixed in methanol to yield concentrations of 5, 200, 15 and 20 μg/mL. *Rhodiola crenulata* was pulverized and filtered through a 40-mesh sieve. Powder (0.5 g) was extracted with 10 mL of methanol. The extract was then filtered through a 0.22 μm filter and dissolved in five methanol to yield a concentration of 12.5 mg/mL.

### Chromatography conditions

The contents of Sal, rhodiosin, tyrosol and *p*-hydroxybenzyl alcohol from *Rhodiola crenulata* were analysed using an Agilent 1290 UHPLC instrument with an autosampler (G4226A), diode array detector (G4212A), quaternary pump (G4220A) and column compartment (G1316C). UHPLC parameters were as follows: samples were separated using a CAPCELL PAK C18 (4.6 mm × 250 mm, 5 μm, Shiseido, Chuo City, Japan) at a temperature of 40 °C, and a detection wavelength of 275 nm. The mobile phase consisted of water-0.1% formic acid (A) and acetonitrile (B) at a flow rate of 1.0 mL/min, gradient elution (v/v): 0–10 min, 9–10% B; 10–10.9 min, 10–12% B; 10.9–20 min, 12–12% B; 20–40 min, 12–22% B; 40–60 min 22–22% B. The injection volume was 5 μL. Data were processed using the MassHunter Workstation software (version B.07.00, Agilent Technologies, Boston, MA).

### Cell culture

The microglial cell line (BV-2) and pheochromocytoma cell line (PC-12) were purchased from the Type Culture Collection of the Chinese Academy of Sciences (Shanghai, China). The cells were cultured in Dulbecco’s modified Eagle medium (DMEM)/F12 medium containing 10% foetal bovine serum, penicillin/streptomycin and high-glucose DMEM containing 10% neonatal bovine serum and penicillin/streptomycin. Before use, the cells were maintained in a CO_2_ humidified incubator (37 °C, 5% CO_2_) (Sanyo, Osaka, Japan). The cell culture medium was changed every day during culture, and the cells were passaged at ∼80% confluence.

### Induction of hypoxia

A modular incubator chamber (Brincubator, MIC-101, Billups-Rothenberg, Del Mar, CA) was used to mimic the hypoxic culture conditions (5% O_2_, 5% CO_2_ and 90% N_2_) in glucose-free medium for oxygen-glucose deprivation cell models. For normoxia, cells were cultured in a normal environment (21% O_2_, 5% CO_2_ and 74% N_2_).

### Cell proliferation testing

BV-2 and PC-12 cells were seeded into 96-well plates at a density of 1 × 10^4^ cells/100 μL for 24 h. Then, they were treated with Sal (66.6, 133.2, 199.8, 266.4, 333.0, 666.0, 1332, 2664, 5328 and 10,656 μM), rhodiosin (5, 10, 25, 50, 100, 200, 400, 800 and 1600 μM), tyrosol (0.13, 0.26, 0.52, 1.04, 2.08, 4.16, 8.32 and 16.64 mM) and *p*-hydroxybenzyl alcohol (0.1, 0.5, 1, 5, 10, 25, 50 and 100 mM) for 24 h. Cells were then treated with Sal (10 μM), rhodiosin (10 μM), tyrosol (10 μM), *p*-hydroxybenzylalcohol (10 μM) or deferoxamine (DFO, 100 μM, solution in DMSO) for 1 h, prior to hypoxic incubation for 3 h. Cells were treated with vehicle, Sal (0.08, 0.016, 0.4, 2, 10, 50, 100 and 200 μM) for 4 h to detect the toxic effects of Sal. Cell proliferation was tested by adding 10 μL of CCK-8 per well for 2 h. The absorbance was measured using a multimode plate reader at 450 nm (PerkinElmer EnSpire, Waltham, MA).

### Western blotting

Lysates from cells were prepared in RIPA lysis buffer containing protease inhibitors and then measured using a BCA Protein Assay Kit (BestBio, Shanghai, China). Lysates (40 μg) were loaded on SDS-PAGE gels for electrophoresis and then transferred to a polyvinylidene fluoride (PVDF) membrane (Merck Millipore, Darmstadt, Germany) at 200 mA for 2 h. After that, the PVDF membranes were blocked with non-fat dry milk in tris-buffered saline for 2 h at room temperature and probed with Hif-1α or β-actin overnight at 4 °C. Membranes were then incubated with anti-rabbit IgG and HRP-linked antibodies for 1 h at room temperature. Afterward, the blots on the membranes were detected with ECL reagent (Merck Millipore, Darmstadt, Germany) using a chemiluminescence imaging system (ChemiScope Mini, Shanghai, China).

### Molecular docking

Two-dimensional structures of Sal, rhodiosin, tyrosol and *p*-hydroxybenzyl alcohol were obtained using ChemDraw. Ligand preparation was performed using Chem3D by energy minimization using an MMP force field. The three-dimensional structure of HIF-1α (PDBID; 4H6J), with a resolution of 1.52 Å, was obtained from the Protein Data Bank database (http://www.rcsb.org/structure/4H6J). Remove the solvent and organic matter using PyMOL. Then, hydrogen was added to the protein using AutoDock Tools. A grid box with grid points (96, 96, 108) and spacing of 0.375 was centred (12.214, −14.282, −21.338) on the given co-crystallized ligand. Molecular docking analysis was performed via AutoDock4. In the dockings, the genetic algorithm was chosen as the search parameter. Visualization was performed using the Discovery Studio program.

### Ribonucleic acid (RNA) analysis by quantitative real-time reverse transcription-polymerase chain reaction (qRT-PCR)

To investigate the gene expression of Hif-1α in BV-2 and PC-12 cells stimulated by Sal, rhodiosin, tyrosol, hydroxybenzylalcohol or DFO for 4 h, qRT-PCR was performed. BV-2 or PC-12 cells were seeded into six-well plates at a density of 6 × 10^5^ or 8 × 10^5^ cells per well, respectively. Total RNA was extracted from BV-2 and PC-12 cells using the TRIzol reagent (Thermo Scientific, Waltham, MA). Total RNA samples (1 μg) were reverse transcribed into cDNA with HiScript^®^ III RT SuperMix for qPCR (Vazyme Biotech Co., Ltd., Nanjing, China) and amplified using ChamQ Universal SYBR qPCR Master Mix (Vazyme Biotech Co., Ltd., Nanjing, China) with a hot denaturation for 30 s at 95 °C. Then, fluorescence intensity was recorded for 40 cycles at 95 °C for 10 s and annealed at 60 °C for 30 s. Glyceraldehyde 3-phosphate dehydrogenase (GAPDH) was used as a housekeeping gene to normalize the expression of Hif-1α. Relative expression was calculated using the 2^–ΔΔCT^ analysis.

1. Hif-1α, forward: 5′-TTCTCCAAGCCCTCCAAGTATGA-3′,
2. reverse: 5′-GCCACTGTATGCTGATGCCTTAG-3′,
3. GAPDH, forward: 5′-CGGAGTCAACGGATTTGGTCGTAT-3′, reverse: 5′-AGCCTTCTCCATGGTGGTGAAGAC-3′.

### Measurement of lactate

BV-2 or PC-12 cells were seeded into 12-well plates at a density of 2 × 10^5^ cells/mL for 24 h. The culture medium was collected at 4 h after treatment with vehicle, Sal (1, 10 and 100 μM) or DFO (100 μM) for quantification of lactate using a Lactate Colorimetric Assay kit (BioVision, Shanghai, China) following the manufacturer’s instructions. The absorbance was measured using a multimode plate reader at 450 nm (PerkinElmer, Waltham, MA).

### Measurement of the extracellular acidification rate (ECAR) and oxygen consumption rate (OCR)

The ECAR and OCR were measured using a Seahorse XF24 analyser (Seahorse Bioscience, Boston, MA) according to the manufacturer’s instructions. Plate BV-2 cells at 8 × 10^4^ cells/100 μL and PC-12 cells at 6 × 10^4^ cells/100 μL previously in the Seahorse XF Microplate, respectively, and treated with vehicle, Sal (1, 10 and 100 μM) or DFO (100 μM). The sensor cartridge was hydrated in a Seahorse XF Calibrant and incubated overnight (37 °C, CO_2_-free). For ECAR measurements, the assay medium of PC-12 was prepared by XF base medium with 4 mM glutamine, and BV-2 was prepared by XF base medium with 2.5 mM glutamine. For OCR measurements, the assay medium of BV-2 (1 mM pyruvate, 2.5 mM glutamine and 17 mM glucose) and PC-12 (XF base medium containing 1 mM pyruvate, 4 mM glutamine and 25 mM glucose) were prepared immediately before assay.

### Animal experiment

Forty male C57BL/6J mice were obtained from the Beijing Vital River Laboratory Animal Technology Co., Ltd. (Beijing, China), certification no. SCXK (Jing) 2016-0006 weighing 22 ± 10 g. The mice were maintained in accordance with the Guidelines for the Care and Use of Laboratory Animals formulated by the Ministry of Science and Technology of China. All procedures were approved by the Animal Ethical and Welfare Committee of Beijing University of Chinese Medicine (approval number: BUCM-4-2021082005-3050). All mice were housed five per cage at a temperature of 21 °C with a 12 h light-dark cycle (lights off at 07:00 h) and at a relative humidity of 40–50% and had free access to standard mice chow and water. The mice were acclimatized to their surroundings for one week and were randomly divided into four groups (*n* = 10/group): control group (normal saline, 0.2 mL i.p.), SAL-L group (25 mg/kg i.p.), SAL-M group (50 mg/kg i.p.) and SAL-H group (100 mg/kg i.p.). The mice were treated with normal saline, SAL-L, SAL-M or SAL-H daily for three days. After the last treatment, the brain of each mouse was isolated after the mice were intracardially perfused with 60 mL of 0.9% normal saline.

### Statistical analysis

Data processing and statistical analysis were performed using GraphPad Prism 6 (La Jolla, CA) with a one-way analysis of variance (ANOVA) using Tukey’s *post hoc* test. All data are expressed as the mean ± SEM. A *p* value of less than 0.05 was considered statistically significant for all tests.

## Results

### Time threshold to mild hypoxia on the proliferation of BV-2 cells and PC-12 cells

**Figure 1.**
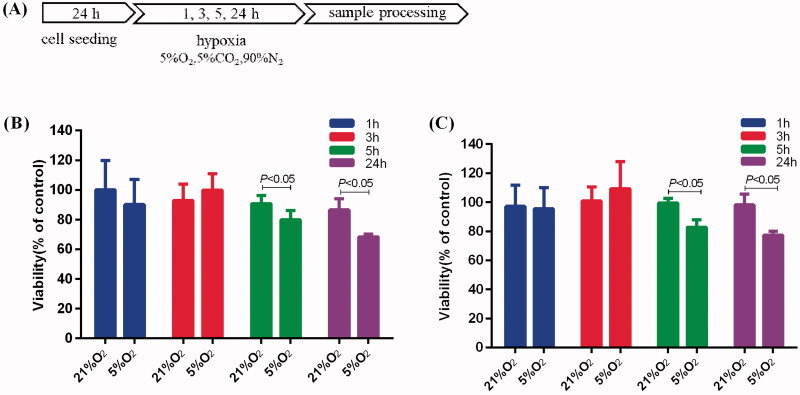
Effects of mild hypoxia on the proliferation of BV-2 and PC-12 cells. (A) Schematic illustration of BV-2 and PC-12 cells induction of hypoxia protocol. (B, C) Effects of mild hypoxia on the proliferation of BV-2 cells (B) and PC-12 cells (C) with 5% O_2_. The cell viability was evaluated using a CCK-8 assay (*n* = 6). Data represent the mean ± SEM and are representative of triplicate experiments (one-way ANOVA).

For the induction of mild hypoxia, BV-2 and PC-12 cells were treated with 5% O_2_ for 1, 3, 5 and 24 h, as shown schematically in Figure 1(A). To investigate the effect of mild hypoxia on proliferation, we assessed the proliferation of BV-2 and PC-12 cells after induction of hypoxia with 5% O_2_. BV-2 cells cultured with 5% O_2_ for 5 h and 24 h exhibited 11% and 18% inhibition, respectively (Figure 1(B)). PC-12 cells exhibited 17% and 21% inhibition, respectively (Figure 1(C)). However, hypoxia with 5% O_2_ for 1 h and 3 h had no effect on the proliferation of BV-2 and PC-12 cells.

### BV-2 were sensitive to mild hypoxia

**Figure 2.**
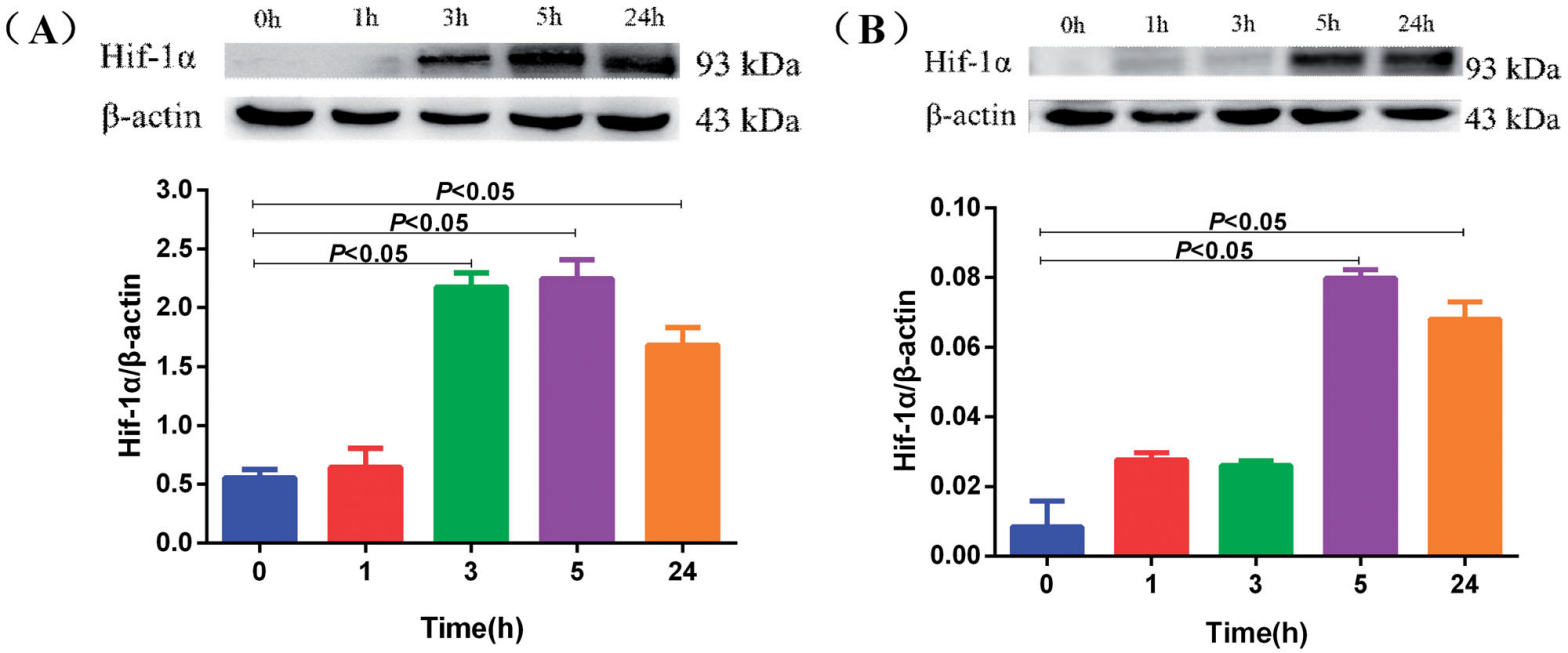
Effects of mild hypoxia on the Hif-1α protein of BV-2 and PC-12 cells. Hif-1α protein expression was measured by Western blotting in BV-2 cells (A) and PC-12 cells (B) treated with 5% O_2_ for 1, 3, 5 and 24 h. β-actin served as a loading control. Data are shown as the mean ± SEM and are representative of triplicate experiments (one-way ANOVA).

We chose DFO as a positive control, which mimics hypoxic conditions because of inducing Hif-1α. We investigated the protein expression of Hif-1α. Western blotting results showed that induction with 5% O_2_ for 3 h increased Hif-1α protein expression in BV-2 cells (Figure 2(A)) and that induction with 5% O_2_ for 5 h increased Hif-1α protein expression in PC-12 cells (Figure 2(B)). These data suggest that BV-2 cells were more sensitive to mild hypoxia than PC-12 cells after induction with 5% O_2_. Therefore, we used 3 h for mild hypoxia in the subsequent experiments.

### Compound-target interactions for the compounds with Hif-1α

**Figure 3.**
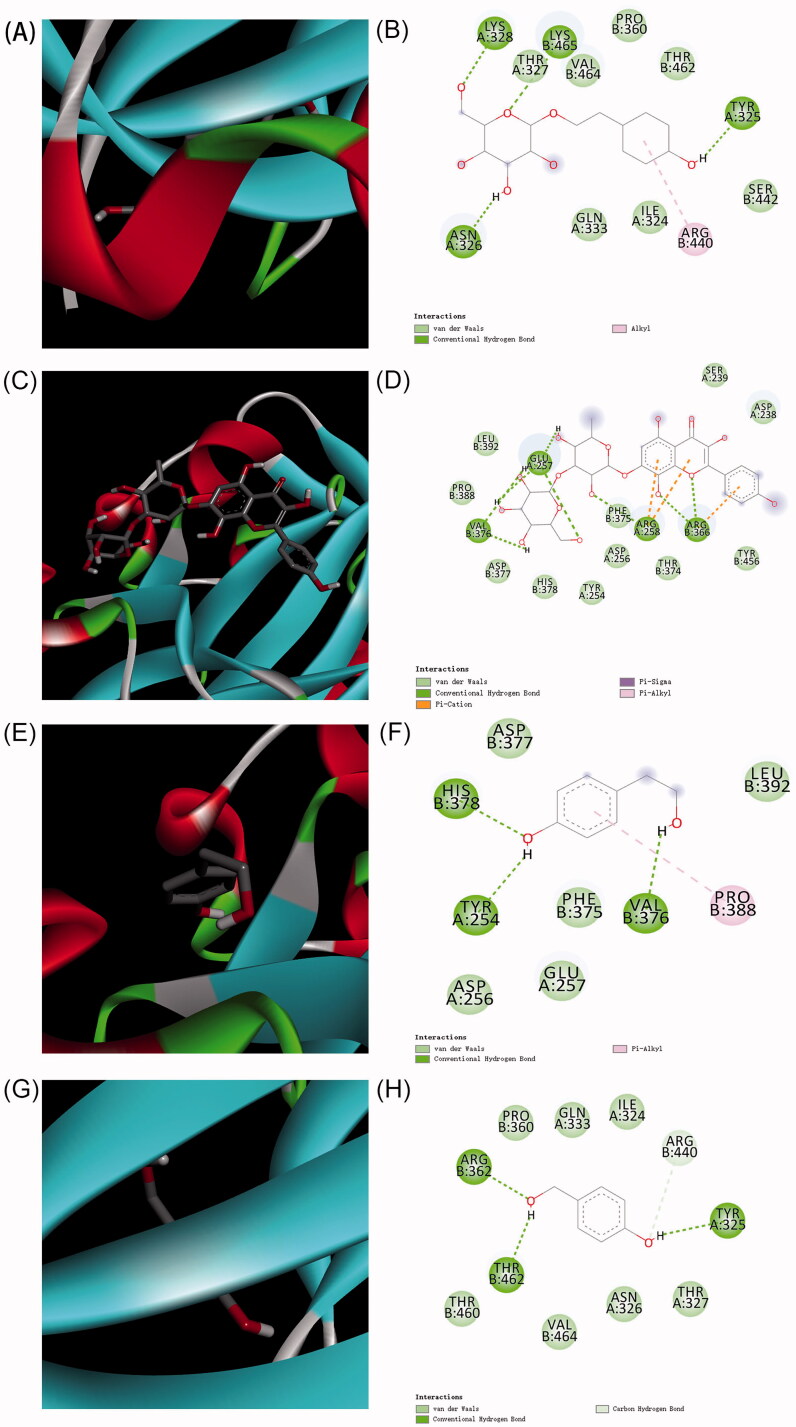
The predicted interactions between Hif-1α with salidroside, rhodiosin, tyrosol and *p*-hydroxybenzyl alcohol. (A, B) Salidroside, (C, D) rhodiosin, (E, F) tyrosol and (G, H) *p*-hydroxybenzyl alcohol.

The contents of Sal, rhodiosin, tyrosol and *p*-hydroxybenzyl alcohol were 13.144, 1.166, 0.898 and 0.506 mg/g, respectively (Figure S1, Table S1), which are the active functional ingredients of *Rhodiola crenulata*. AutoDock was used to determine the binding patterns of small molecules to proteins. Interestingly, the docking results showed that Sal, rhodiosin, tyrosol and *p*-hydroxybenzyl alcohol had a binding energy of −5.24, −5.20, −4.89 and −4.88 kcal/mol, respectively, suggesting that Sal exhibited a stronger binding ability to Hif-1α (Table 1). Next, the conformation of the interactions between these compounds and 4H6J was analysed using Discovery Studio. An interaction analysis revealed that Sal had hydrogen-bonding interactions with 4H6J via residues ASN326, TYR325, LYS328 and LYS465 in different directions (Figure 3(A,B)). Furthermore, we found van der Waals forces via residues GLN333, ILE324, SER442, THR462, PRO360, VAL464, THR327 and Alktl via ARE440. In addition, rhodiosin interacted via hydrogen bonds with VAL376, GLU257, ARG366, ARG258, van der Waals forces with ASP377, HIS378, TYR254, ASP256, THR374, TYR456, ASP238, SER239, PHE375, LEU392, PRO388, Pi-Cation with ARG366 and ARG258, Pi-Sigma, and Pi-Alktl (Figure 3(C,D)). Tyrosol interacted via hydrogen bonds with TYR254, VAL376, HIS378, van der Waals forces with ASP377, ASP256, GLU257, PHE375, LEU392, and Pi-Alktl with PRO388 (Figure 3(E,F)). *p*-Hydroxybenzyl alcohol interacted via hydrogen bonds with ARG362, THR462, TYR325, ARG440, and van der Waals forces with THR460, VAL464, ASN326, THR327, ILE324, GLN333 and PRO360 (Figure 3(G,H)). The molecular interactions showed that Sal, rhodiosin, tyrosol and *p*-hydroxybenzyl alcohol bond to the active sites of Hif-1α, based on the hydrogen bonds and van der Waals forces. Collectively, our results showed that the complex formed by hif-1α with Sal was more stable than rhodiosin, tyrosol and *p*-hydroxybenzyl alcohol, suggesting that Sal exhibited a stronger binding ability to Hif-1α.

**Table 1.** Binding energy of between Hif-1α with salidroside, rhodiosin, tyrosol and *p*-hydroxybenzyl alcohol.

Ligand	Binding energy (kcal/mol)
Salidroside	–5.24
Rhodiosin	–5.2
Tyrosol	–4.89
*p*-Hydroxybenzyl alcohol	–4.88

### Salidroside blocked Hif-1α degradation in BV-2 and PC-12 cells under normoxia

**Figure 4.**
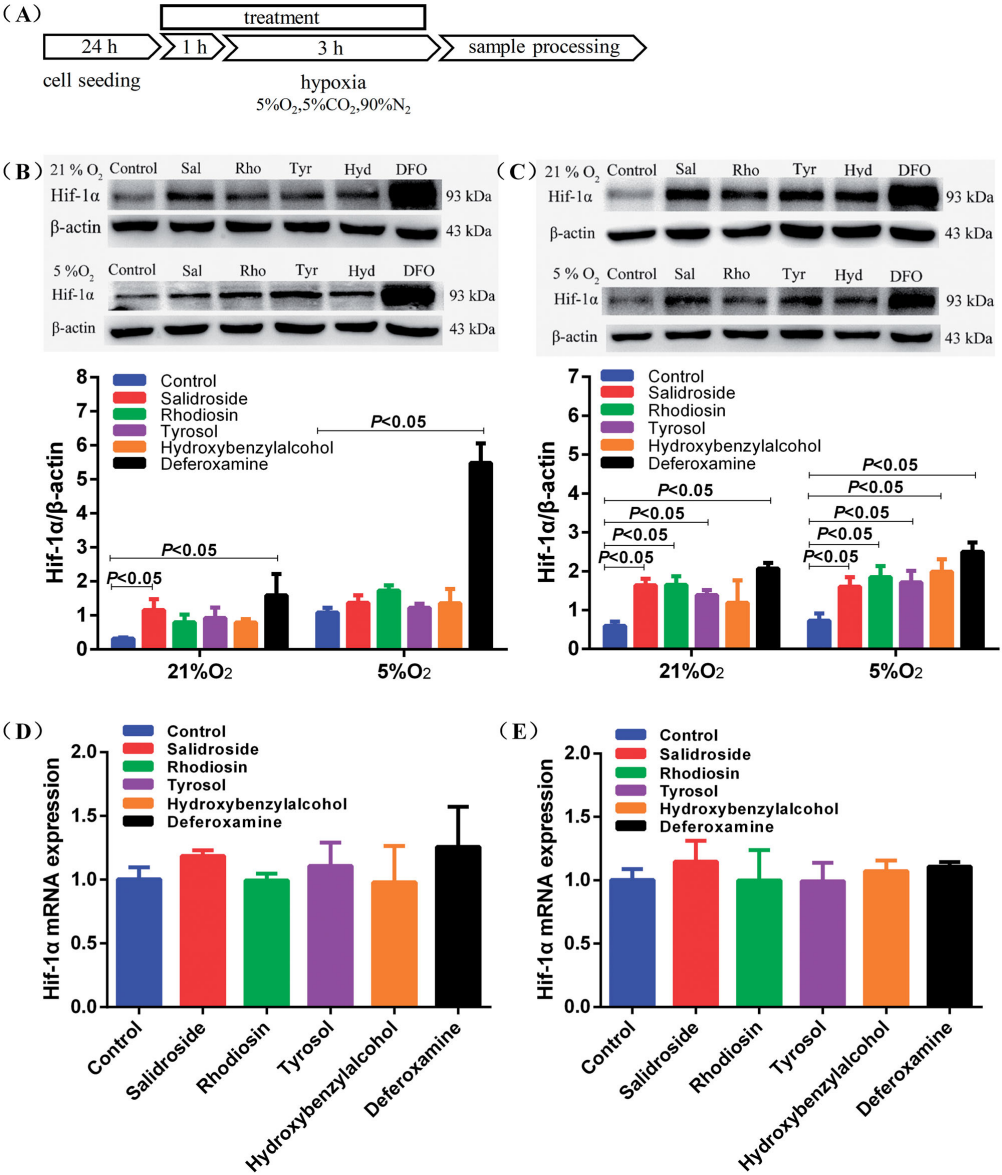
Salidroside blocked Hif-1α degradation in BV-2 and PC-12 cells. (A) Schematic illustration of BV-2 and PC-12 cells treatment protocol. (B, C) Hif-1α protein expression was measured by Western blotting in BV-2 cells (B) and PC-12 cells (C) treated with salidroside (Sal, 10 μM), rhodiosin (Rho, 10 μM), tyrosol (Tyr, 10 μM), *p*-hydroxybenzyl alcohol (Hyd, 10 μM) or deferoxamine (DFO, 100 μM) under normoxia (21% O_2_, 5% CO_2_) or mild hypoxia (5% O_2_, 5% CO_2_). β-actin served as a loading control. (D, E) Relative mRNA expression of Hif-1α in BV-2 cells (D) and PC-12 cells (E) treated with salidroside, rhodiosin, tyrosol, *p*-hydroxybenzyl alcohol or desferrioxamine under normoxia for 4 h. GAPDH was the housekeeping control. Data are shown as the mean ± SEM and are representative of triplicate experiments (one-way ANOVA).

To verify the relationship between the compositions of *Rhodiola crenulata* and the Hif-1α degradation pathway, we sought to determine the effects of Sal, rhodiosin, tyrosol and *p*-hydroxybenzyl alcohol on the protein expression of Hif-1α in BV-2 and PC-12 cells under normoxia or hypoxia. The treatment protocol is shown schematically in Figure 4(A). The IC_50_ values of Sal, rhodiosin, tyrosol and *p*-hydroxybenzyl alcohol for BV-2 were 1.93 ± 0.34 mM (Figure S2A), 959.74 ± 10.24 μM (Figure S2B), 7.47 ± 1.03 mM (Figure S2C) and 8.42 ± 1.63 mM (Figure S2D), and for PC-12 were 6.89 ± 0.57 mM (Figure S2A), 159.28 ± 8.89 μM (Figure S2B), 8.65 ± 1.20 mM (Figure S2C) and 8.64 ± 1.42 mM (Figure S2D), respectively. Notably, Sal treatment dramatically increased the protein expression of Hif-1α under normoxia in BV-2 and PC-12 cells. Salidroside, rhodiosin, tyrosol and *p*-hydroxybenzyl alcohol reduced Hif-1α degradation in BV-2 (3.7-, 2.5-, 2.9- and 2.5-fold) and PC-12 cells (2.8-, 2.8-, 2.3- and 2.0-fold) under normoxia. The protein level of Hif-1α was higher after treatment with Sal than with rhodiosin, tyrosol and *p*-hydroxybenzyl alcohol treatments in BV-2 and PC-12 cells (Figure 4(B,C)). We also found that Sal, rhodiosin, tyrosol and *p*-hydroxybenzyl alcohol did not affect the Hif-1α protein in BV-2 cells under hypoxic conditions compared with the control. However, they successfully enhanced the expression of Hif-1α protein in PC-12 cells because hypoxic treatment for 3 h did not upregulate Hif-1α protein expression (Figure 4(B,C)). Sal, rhodiosin, tyrosol and *p*-hydroxybenzyl alcohol did not affect the mRNA levels of Hif-1α under normoxia in BV-2 and PC-12 cells (Figure 4(D,E)). These data suggest that Sal dampened Hif-1α degradation in BV-2 and PC-12 cells under normoxia.

### Salidroside induced metabolic reprogramming from oxidative phosphorylation to glycolysis

**Figure 5.**
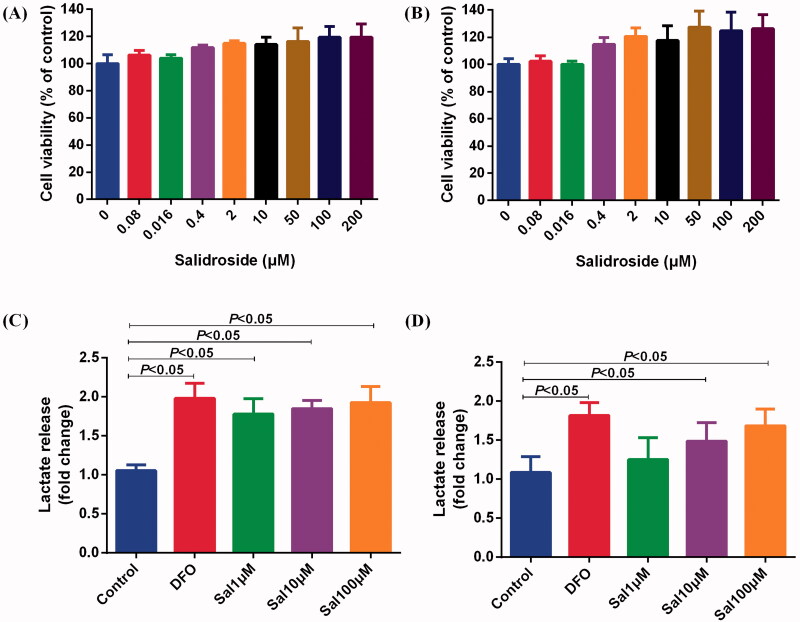
Salidroside increased the secretion of lactic acid in BV-2 and PC-12 cells. (A, B) Effects of different concentrations of salidroside on the proliferation of BV-2 cells (A) and PC-12 cells (B). The cell viability was evaluated using a CCK-8 assay (*n* = 6 per group). (C, D) The secretion of lactate in BV-2 cells (C) and PC-12 cells (D) treated with vehicle, salidroside (Sal, 1, 10 and 100 μM), or deferoxamine (DFO, 100 μM). The lactate release was evaluated using a Lactate Colorimetric Assay kit (*n* = 6 per group). Data are shown as the mean ± SEM and are representative of triplicate experiments (one-way ANOVA).

Glycolysis enables cell proliferation, migration, cytokine secretion and phagocytosis because the glucose metabolism rate in glycolysis is 10–100 times faster than that of oxidative phosphorylation (Baik et al. 2019). Cellular energy metabolic pathways in the central nervous system may be closely linked to their effector function. To determine whether Sal is involved in metabolic reprogramming, we assessed the status of glycolysis and mitochondrial respiration in BV-2 and PC-12 cells after treatment with Sal. Cell proliferation analyses showed that Sal did not show any toxic effects in BV-2 and PC-12 cells at a concentration of 200 μM (Figure 5(A,B)). After Sal treatment for 4 h, the lactic acid in the supernatant was increased compared with the control, which indicated Sal induced glycolysis in BV-2 and PC-12 cells (Figure 5(C,D)).

**Figure 6.**
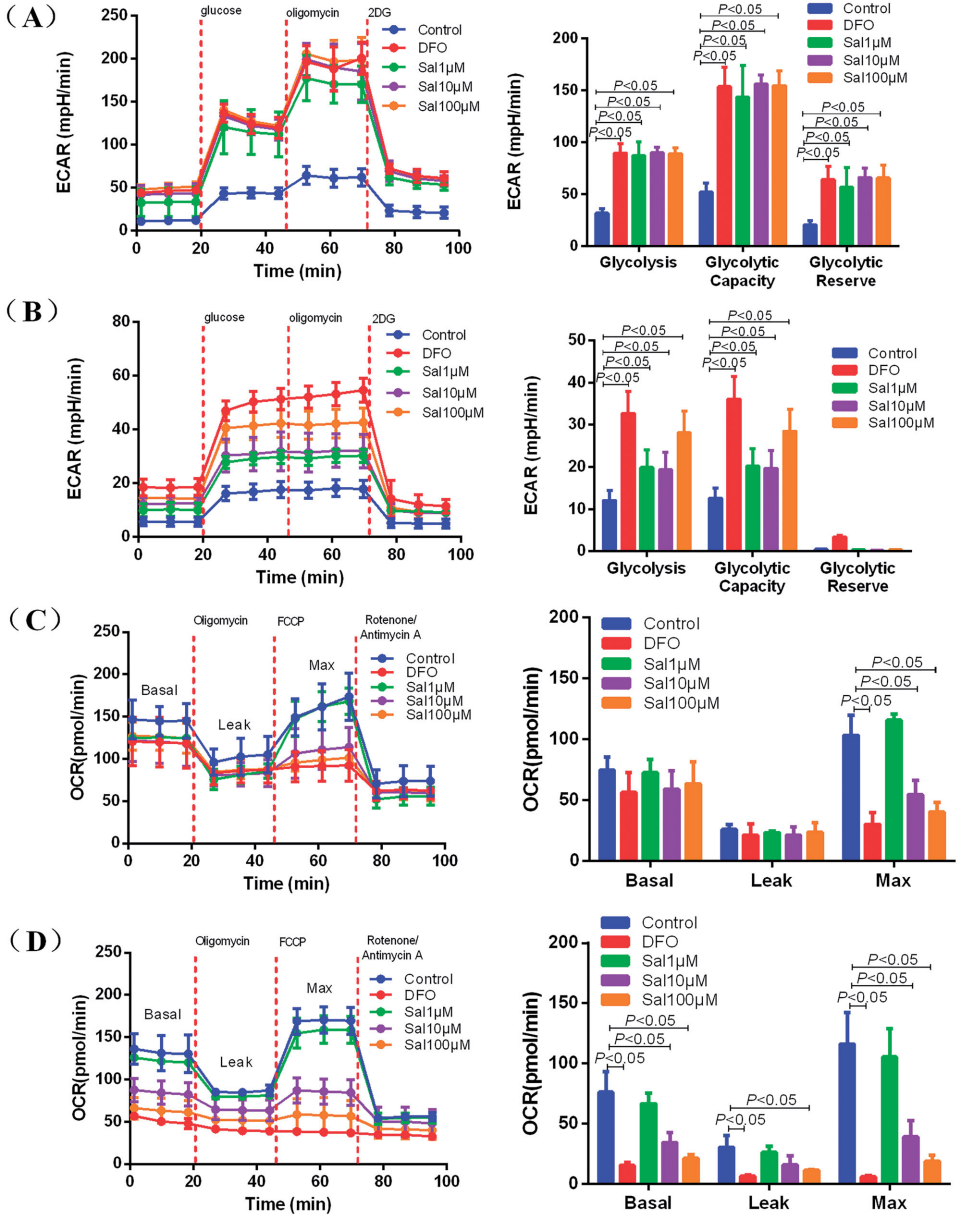
Salidroside induced metabolic reprogramming from oxidative phosphorylation to glycolysis in BV-2 and PC-12 cells. (A, B) Glycolysis determined by measuring ECAR in BV-2 cells (A) (*n* = 4 per group) and PC-12 cells (B) (*n* = 4 per group) treated with vehicle, salidroside (Sal, 1, 10 and 100 μM), or deferoxamine (DFO, 100 μM). (C, D) Oxidative phosphorylation determined by measuring OCR in BV-2 cells (C) (*n* = 4 per group) and in PC-12 cells (D) (*n* = 4 per group) treated with vehicle, salidroside (1, 10 and 100 μM), or DFO (100 μM). Data are shown as the mean ± SEM and are representative of triplicate experiments (one-way ANOVA).

The ECAR was measured as a measure of glycolysis after treatment with glucose (a saturating concentration of glucose), oligomycin (an ATP synthase inhibitor) and 2-deoxy-D-glucose (2-DG) (a glucose analogue). The results revealed that the glycolytic capacity level was increased by 91.5%, 104.0% and 102.4% after exposure to Sal at doses of 1, 10 and 100 μM for 4 h in BV-2 cells compared with the control (Figure 6(A)). The glycolytic capacity was increased by 7.6%, 7.1% and 15.9% at 1, 10 and 100 μM of Sal for 4 h in PC-12 cells (Figure 6(B)). These data indicate a shift towards glycolytic metabolism in BV-2 and PC-12 cells.

As we observed that Sal regulated glycolysis levels, we hypothesized that Sal also regulated oxidative phosphorylation efficiency. To confirm this, the changes in the OCR were measured, an indicator of mitochondrial respiration after treatment with oligomycin (an ATP synthase inhibitor), cyanide p-trifluoromethoxyphenyl-hydrazone (FCCP) (H^+^ ionophore), and a mixture of rotenone and antimycin A (electron-transport chain inhibitor), to assess mitochondrial function. The results implied that Sal treatment at doses of 10 and 100 μM significantly diminished the maximal respiratory capacity of mitochondria in BV-2 and PC-12 cells compared with the control (Figure 6(C,D)). These data demonstrate that Sal increased glycolysis while attenuating oxidative phosphorylation, suggesting that metabolic reprogramming towards glycolysis is critical for the prevention of AMS by Sal.

### Salidroside induced glycolytic metabolism in mice

**Figure 7.**
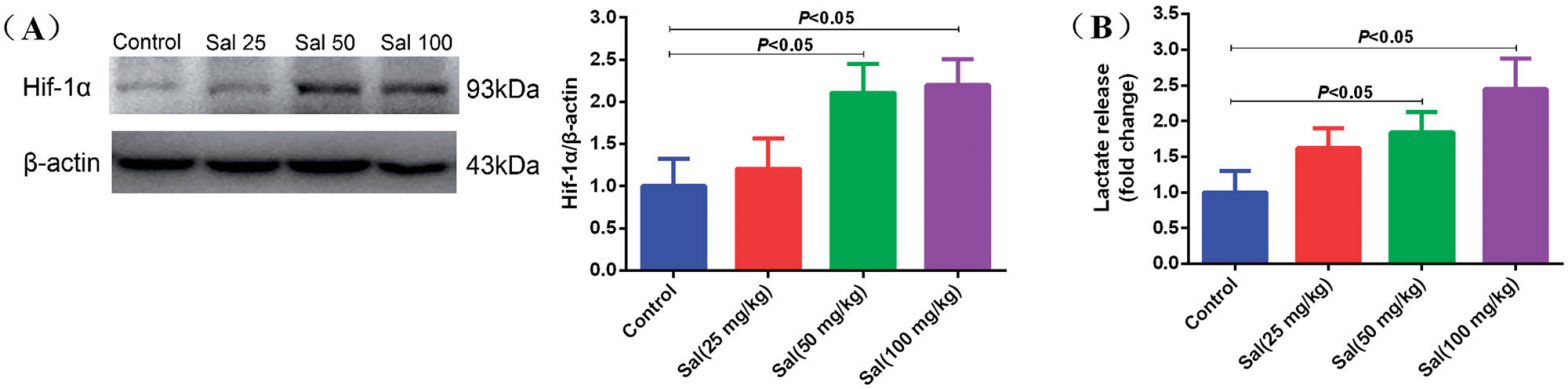
Salidroside induced glycolytic metabolism in mice. (A) Hif-1α protein expression was measured by Western blotting in mice after salidroside treatment (Sal, 25, 50 and 100 mg/kg). β-actin served as a loading control. (B) The secretion of lactate in the brain tissue of mice treated with vehicle, salidroside. The lactate release was evaluated using a Lactate Colorimetric Assay kit (*n* = 10 per group). Data are shown as the mean ± SEM and are representative of triplicate experiments (one-way ANOVA).

To better verify the mechanisms of the central nervous system functions induced by Sal against AMS, we performed animal experiments to detect the protein expression level of Hif-1α and the secretion of lactate in the brain of mice. After administering Sal at 50 and 100 mg/kg i.p. for three days, Sal led to a significant upregulation of Hif-1α protein expression compared with the control (Figure 7(A,B)). In addition, the lactic acid in the brain tissue increased after Sal treatment, suggesting that Sal induced glycolytic metabolism *in vivo*.

## Discussion

Taking *Rhodiola crenulata* in advance of exposure to high altitudes can prevent and treat AMS. Our study revealed that cellular metabolism was reprogrammed to activate the compensatory response of the central nervous system after the administration of Sal under normoxia. Our results demonstrated the following: (1) microglia were more sensitive to mild hypoxia than neurons; (2) Sal dampened Hif-1α degradation under normoxia; and (3) Sal-triggered metabolic reprogramming from oxidative phosphorylation to glycolysis. Our results provide strategies for the protection of AMS from the perspective of cellular metabolism.

Hif-1α serves as the core initiator molecule for the cells to initiate the hypoxic protection reaction, which mediates the basic oxygen sensing mechanism under hypoxic conditions (Semenza 2004). During transient mild hypoxia, intracellular accumulation of Hif-1 strongly protects against hypoxic injury. Hypoxic preconditioning can increase cell anoxic tolerance and survival (Wang et al. 2008). In this study, we observed that treatment with 5% O_2_ increased Hif-1α expression in BV-2 cells for 3 h while PC-12 cells for 5 h, indicating that BV-2 cells were more sensitive to low oxygen levels than PC-12 cells. Our results confirmed that microglia responded to mild hypoxia earlier than the neurons.

Microglia and neurons are highly susceptible to hypoxia, which leads to brain cell injury and neuronal death. Many studies suggest that Sal, rhodiosin, tyrosol and *p*-hydroxybenzyl alcohol exert anti-hypoxic pharmacological effects, including neuroprotection and the activation of microglial cells. Our results clearly showed that Sal had a stronger binding ability to Hif-1α with hydrogen-bonding interactions and van der Waals and Alktl forces in different directions. Consistent with these studies, western blotting demonstrated that the protein level of Hif-1α was higher after treatment with Sal than with rhodiosin, tyrosol and *p*-hydroxybenzyl alcohol treatments in BV-2 and PC-12 cells under normoxia or hypoxia. Previous studies have shown that Sal modulates microglial polarization to protect neurons after cerebral ischaemia (Liu et al. 2018). However, Sal did not affect the mRNA levels of Hif-1α under normoxia. Taken together, these data suggest that Sal induces an increase in Hif-1α protein in microglia triggered neuroprotection under normoxia, which might be associated with the prevention of AMS in *Rhodiola crenulata*.

Hif-1 mediates hypoxia and regulates more than 1000 direct transcriptional targets, including metabolic adaptation, angiogenesis, cell cycle and apoptosis (Schodel and Ratcliffe 2019). Moreover, Hif-1α upregulates the transcription of glycolytic genes, resulting in a metabolic switch from oxidative phosphorylation to glycolysis under hypoxia to supply cellular energy demands (Semenza 2011, 2012). Recent studies have revealed that immune cell metabolism reflects their status. In the present study, Sal changed brain energy metabolism in mice and induced microglial and neural metabolic reprogramming. Sal enhanced lactic acid production in BV-2 cells. Furthermore, ECAR increased after exposure to Sal and reached a plateau at a dose of 10 μM, while the maximal respiratory capacity of mitochondria was diminished. These findings indicate that Sal induced metabolic reprogramming towards glycolysis in immune cells, activating inflammation. In addition, we observed metabolic reprogramming towards glycolysis in neurons. Neurons increase glycolysis in the face of acute energy demand, which provides a faster response to energy (Yellen 2018). Glycolytic enzymes located at the plasma membrane and synaptic vesicles are conducive to supply acute ATP to neurons (Ikemoto et al. 2003; Hinckelmann et al. 2016). This might explain why Sal increases glycolysis as the main energy source for neurons in response to stimulation. The mammalian target of the rapamycin (mTOR) pathway senses cellular energy status by regulating glucose metabolism, which regulates the expression of Hif-1α, which serves as the master transcriptional regulator of glycolysis (Cheng et al. 2014). These findings collectively suggest that Sal induced metabolic reprogramming depends on the Hif-1α pathway in the central nervous system, which might be enhanced by the phosphorylation of mTOR. Nevertheless, this speculation requires further investigation. We will consider the cell metabolism of astrocytes in future experiments to better explain the mechanisms of the functions of the central nervous system against AMS.

## Conclusions

**Figure 8.**
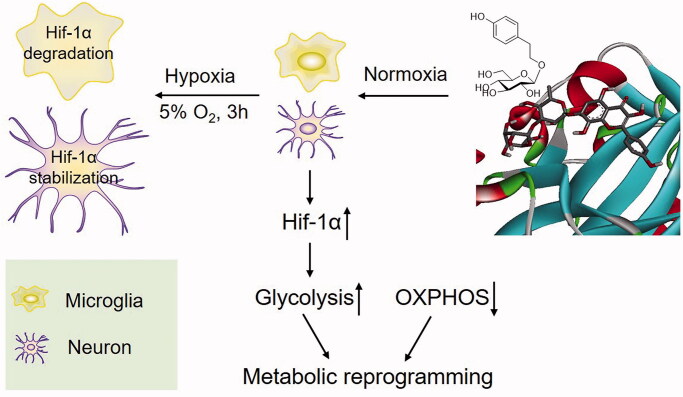
Proposed mechanism for the prevention of salidroside against AMS.

In conclusion (Figure 8), we have demonstrated that microglia respond to mild hypoxia earlier than neurons in the central nervous system. Furthermore, we have confirmed that Sal activates compensatory responses in the mouse brain, activates microglia and improves neural function by dampening Hif-1α degradation under normoxia. The improved endurance performance mechanism of Sal is potentially associated with the orchestration of metabolic reprogramming of neurons and microglia from oxidative phosphorylation to glycolysis.

## Supplementary Material

Supplemental Material
