# Supplementary material for: Salidroside orchestrates metabolic reprogramming by regulating the Hif-1α signalling pathway in acute mountain sickness
Source: Pharm Biol. 2021 Nov 5;59(1):1538–48. doi: 10.1080/13880209.2021.1992449 (PMC8594887; doi:10.1080/13880209.2021.1992449)
Supplement: Supplemental Material [file IPHB_A_1992449_SM3200.docx]

**SUPPLEMENTAl MATERIAL**

**S****alidroside** **orchestrates** **metabolic reprogramming by regulating the Hif-1α signaling pathway in acute mountain sickness**

Xiaoning Yan^a,b^, Jie Liu^b^, Meixia Zhu^a,b^, Lirong Liu^a,b^, Yijun Chen^b^, Yinhuan Zhang^a,b^, Menghan Feng^a,b^, Zhixin Jia^b^, Hongbin Xiao^a,b^*

^a^School of Chinese Materia Medica, Beijing University of Chinese Medicine, Beijing, China; ^b^Research Center of Chinese Medicine Analysis and Transformation, Beijing University of Chinese Medicine, Beijing, China

Correspondence should be addressed to Hongbin Xiao; [hbxiao69@163.com](mailto:hbxiao69@163.com); School of Chinese Materia Medica; Research Center of Chinese Medicine Analysis and Transformation, Beijing University of Chinese Medicine, No. 166 Daxuedong Road, Beijing 100029, People’s Republic of China

**Materials and methods**

***Preparations of*** ***reference substances and samples***

Reference substances of *p*-hydroxybenzyl alcohol, salidroside, tyrosol, and rhodiosin were mixed in methanol to yield concentrations of 5, 200, 15, and 20 μg/mL. *Rhodiola crenulata* was pulverized and filtered through a 40-mesh sieve. Powder (0.5 g) was extracted with 10 mL of methanol. The extract was then filtered through a 0.22 μm filter and dissolved in five methanol to yield a concentration of 12.5 mg/mL.

***Chromatography conditions***

The contents of salidroside, rhodiosin, tyrosol, and *p*-hydroxybenzyl alcohol from *Rhodiola crenulata* were analyzed using an Agilent 1290 UHPLC instrument with an autosampler (G4226A), diode array detector (G4212A), quaternary pump (G4220A), and column compartment (G1316C). UHPLC parameters were as follows: samples were separated using a CAPCELL PAK C18 (4.6 mm × 250 mm, 5 μm, Shiseido) at a temperature of 40°C, and a detection wavelength of 275 nm. The mobile phase consisted of water-0.1% formic acid (A) and acetonitrile (B) at a flow rate of 1.0 mL/min, gradient elution (v/v): 0-10 min, 9%-10% B; 10-10.9 min, 10%-12% B; 10.9-20 min, 12%-12% B; 20-40 min, 12-22% B; 40-60 min 22-22% B. The injection volume was 5 μL. Data were processed using the MassHunter Workstation software (version B.07.00, Agilent Technologies, USA).

***Cell*** ***proliferation testing***

BV-2 and PC-12 cells were seeded into 96-well plates at a density of 1 × 10^4^ cells/100 μL for 24 h. Then, they were treated with salidroside (66.6, 133.2, 199.8, 266.4, 333.0, 666.0, 1332, 2664, 5328, 10656 μM), rhodiosin (5, 10, 25, 50, 100, 200, 400, 800, 1600 μM), tyrosol (0.13, 0.26, 0.52, 1.04, 2.08, 4.16, 8.32, 16.64 mM), and *p*-hydroxybenzyl alcohol (0.1, 0.5, 1, 5, 10, 25, 50, 100 mM) for 24 h. Cell proliferation was tested by adding 10 μL of CCK-8 per well for 2 h. The absorbance was measured using a multimode plate reader at 450 nm (PerkinElmer EnSpire, USA).

**Table S1**. The contents of salidroside, rhodiosin, tyrosol and, *p*-hydroxybenzyl alcohol from *Rhodiola crenulata*.

| Peak no. | RT (min) | Compound | Formula | Content (mg/g) |
| --- | --- | --- | --- | --- |
| 1 | 6.721 | *p*-hydroxybenzyl alcohol | C_7_H_8_O_2_ | 0.506 |
| 2 | 7.753 | Salidroside | C_14_H_20_O_7_ | 13.144 |
| 3 | 11.102 | Tyrosol | C_8_H_10_O_2_ | 0.898 |
| 4 | 47.932 | Rhodiosin | C_27_H_30_O_16_ | 1.166 |


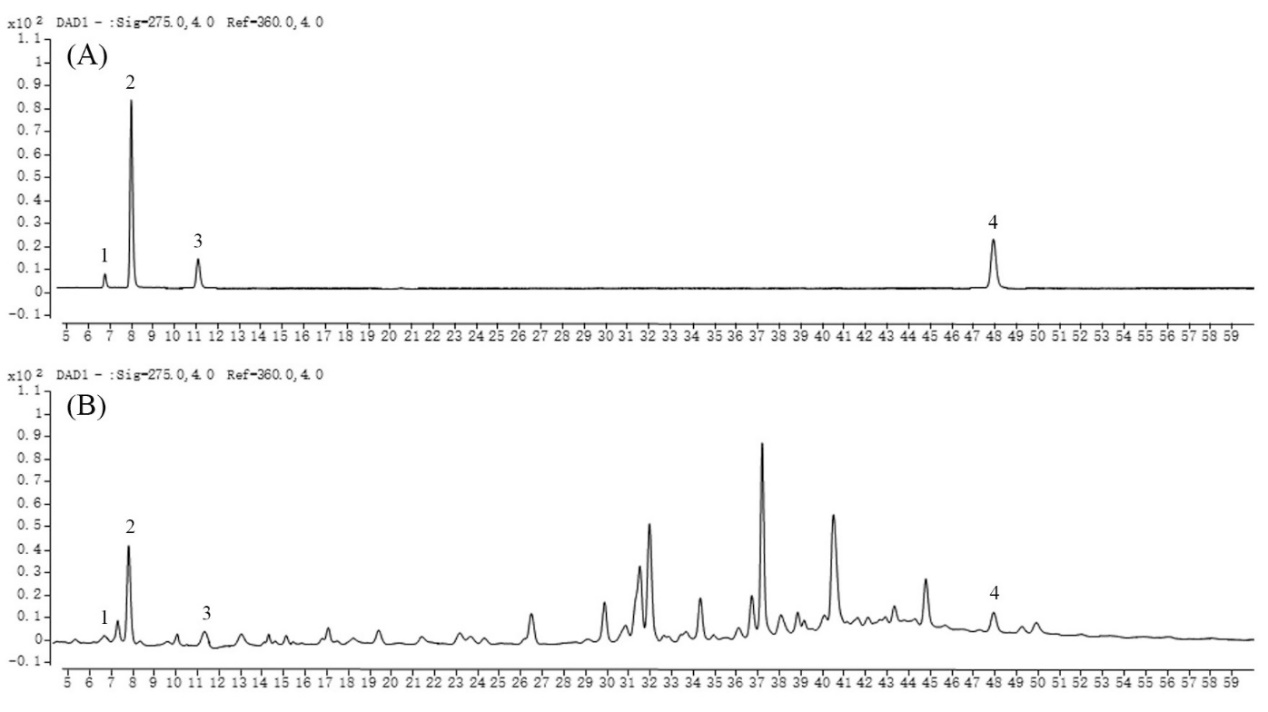


**Figure S1**. UHPLC chromatograms of mixed reference substances (A), and Rhodiola crenulate sample (B). (1) *p*-hydroxybenzyl alcohol; (2) salidroside; (3) tyrosol; (4) rhodiosin.


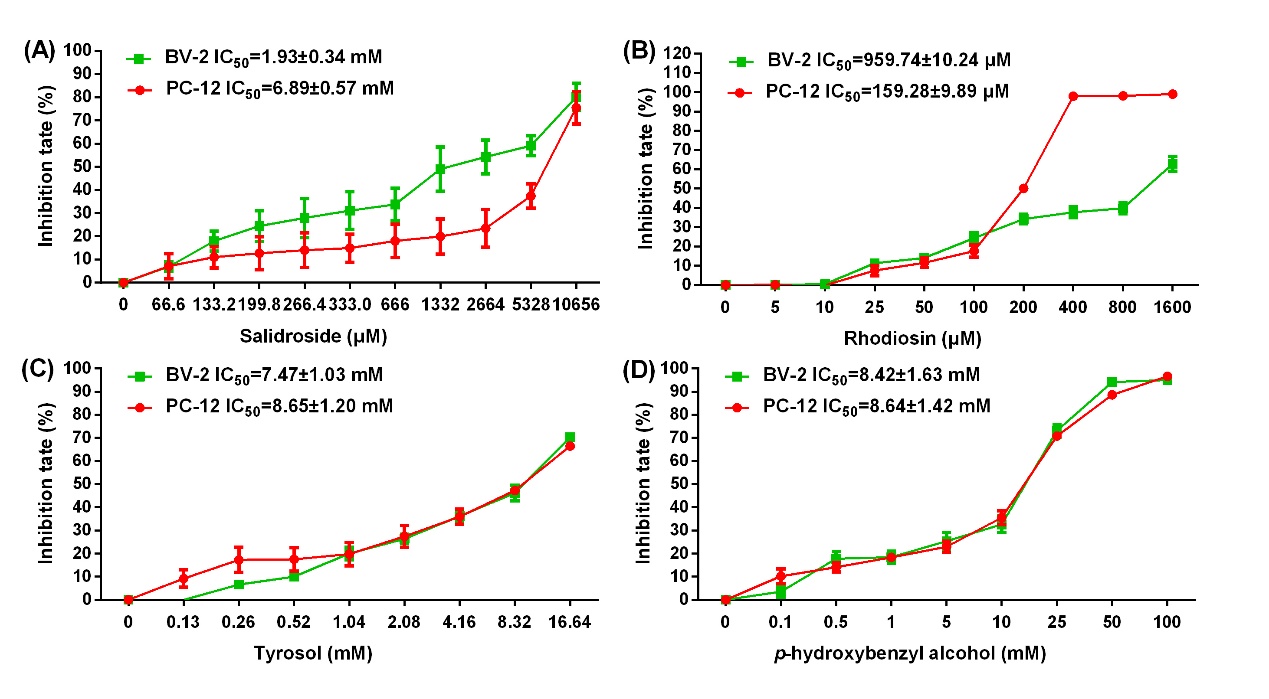


**Figure S2**. Cell viability of BV-2 and PC-12 cells after exposure to salidroside (A), rhodiosin (B), tyrosol (C), and *p*-hydroxybenzyl alcohol (D) for 24 h, respectively. The cell viability was evaluated using a CCK-8 assay (n = 6 per group). Data are expressed as the means of triplicate experiments.
